# Diet-informed metagenomic clocks for aging in the Integrative Longevity Omics Study

**DOI:** 10.1093/geroni/igaf122.4101

**Published:** 2025-12-31

**Authors:** Tanya Karagiannis, Anastasia Leshchyk, Meghan Short, Ye Chen, Sarah Bald, Stacy Andersen, Daniel Segrè, Paola Sebastiani

**Affiliations:** Tufts Medical Center, Boston, Massachusetts, United States; Tufts Medical Center, Boston, Massachusetts, United States; Tufts University School of Medicine, Medford, Massachusetts, United States; Tufts Medical Center, Boston, Massachusetts, United States; Boston University, Boston, Massachusetts, United States; Boston University, Boston, Massachusetts, United States; Boston University, Boston, Massachusetts, United States; Tufts Medical Center, Boston, Massachusetts, United States

## Abstract

Aging is a heterogeneous process that unfolds uniquely across individuals and biological systems. Aging clocks, computational models trained on molecular data to estimate biological age, offer a promising framework for investigating aging biology at the individual level. While most clocks rely on epigenetic, transcriptomic, or proteomic data, the human microbiome remains an underutilized source of aging biomarkers despite its central role in host metabolism, immune regulation, and inflammation, all of which are influenced by diet and linked to aging. To explore microbiome-mediated aging, we developed aging clocks using shotgun metagenomics sequencing data from 267 individuals in the Integrative Longevity Omics cohort, including centenarians and their offspring. These clocks estimate biological age based on taxonomic composition across multiple taxonomic levels. To account for dietary influences, we incorporated total caloric intake and a novel metric developed by our group, the Nutrient Variety Index (NVI), which summarizes the distribution of nutrient groups within a diet. Adjusting for caloric intake and NVIs significantly improved clock performance, suggesting that nutrient composition modulates microbiome aging. This integrative approach enhances biological age estimation and has the potential to uncover microbial signatures of healthy aging. Our findings underscore the importance of diet in shaping aging trajectories and highlight the potential of microbiome-based clocks to uncover nutrition-linked aging pathways.

